# Metagenomic analysis reveals a green sulfur bacterium as a potential coral symbiont

**DOI:** 10.1038/s41598-017-09032-4

**Published:** 2017-08-24

**Authors:** Lin Cai, Guowei Zhou, Ren-Mao Tian, Haoya Tong, Weipeng Zhang, Jin Sun, Wei Ding, Yue Him Wong, James Y. Xie, Jian-Wen Qiu, Sheng Liu, Hui Huang, Pei-Yuan Qian

**Affiliations:** 1 Shenzhen Research Institute and Division of Life Science, The Hong Kong University of Science and Technology, Hong Kong SAR, China; 2 Key Laboratory of Tropical Marine Bio-resources and Ecology, South China Sea Institute of Oceanology, Chinese Academy of Sciences, Guangzhou, China; 3 Department of Biology, Hong Kong Baptist University, Hong Kong SAR, China

## Abstract

Coral reefs are ecologically significant habitats. Coral-algal symbiosis confers ecological success on coral reefs and coral-microbial symbiosis is also vital to coral reefs. However, current understanding of coral-microbial symbiosis on a genomic scale is largely unknown. Here we report a potential microbial symbiont in corals revealed by metagenomics-based genomic study. Microbial cells in coral were enriched for metagenomic analysis and a high-quality draft genome of “*Candidatus* Prosthecochloris korallensis” was recovered by metagenome assembly and genome binning. Phylogenetic analysis shows “*Ca*. P. korallensis” belongs to the *Prosthecochloris* clade and is clustered with two *Prosthecochloris* clones derived from Caribbean corals. Genomic analysis reveals “*Ca*. P. korallensis” has potentially important ecological functions including anoxygenic photosynthesis, carbon fixation via the reductive tricarboxylic acid (rTCA) cycle, nitrogen fixation, and sulfur oxidization. Core metabolic pathway analysis suggests “*Ca*. P. korallensis” is a green sulfur bacterium capable of photoautotrophy or mixotrophy. Potential host-microbial interaction reveals a symbiotic relationship: “*Ca*. P. korallensis” might provide organic and nitrogenous nutrients to its host and detoxify sulfide for the host; the host might provide “*Ca*. P. korallensis” with an anaerobic environment for survival, carbon dioxide and acetate for growth, and hydrogen sulfide as an electron donor for photosynthesis.

## Introduction

Coral reefs are significant marine ecosystems, but they are declining at an alarming rate, primarily due to climate change, ocean acidification, and anthropogenic disturbances^1, 2^. Reef-building corals form symbiotic associations with dinoflagellates of the genus *Symbiodinium* ^3^. These symbiotic algae provide organic nutrients for coral growth and calcification allowing reef formation in nutrient-limited oceans, while they gain inorganic carbon, essential elements and refuge from the host corals in return^3^. In addition to symbiotic algae, highly diverse and abundant prokaryotic microbes have been found in the coral mucus, tissue, and skeleton^4^. Coral hosts can benefit from these microbes through the carbon, nitrogen, and sulfur cycles and from antimicrobial defense, which may be critical in promoting coral growth and preventing disease^5, 6^. The term coral holobiont has been used to decribe the entity consisting of the coral host, *Symbiodinium*, and associated microbes including bacteria, archaea, fungi, viruses, protozoa, and other unknown components^7^. The coral holobiont is a highly dynamic system mediated by complex interactions between the coral host and its members, because of constant exposure to a changing environment. Exploration of potential coral microbial symbiots involved in these complex interactions is crucial to strengthen our understanding of symbiosis in the coral holobiont, contributing to a better coral reef conservation.

Green sulfur bacteria are thought to have an ancient origin on Earth, and are ecologically and biogeochemically important drivers of the carbon, nitrogen, and sulfur cycles^8, 9^. They are mostly found in anoxic aquatic environments with both light and sulfide, performing anoxygenic photosynthesis using electrons generated by sulfur oxidation^10^. Green sulfur bacteria fix carbon dioxide via the reductive tricarboxylic acid (rTCA) cycle but some organic compounds like acetate and pyruvate can be assimilated during mixotrophic growth^9, 11^. As they are able to use nitrogen gas as the sole nitrogen source for growth, they are nitrogen fixers^12^. Green sulfur bacteria are grouped taxonomically under the family *Chlorobiaceae* in the phylum *Chlorobi* ^13^, which are phylogenetically distant from the other phototrophic bacteria in the phyla *Chloroflexi*, *Cyanobacteria*, *Proteobacteria*, and *Firmicutes*. Most identified green sulfur bacteria are free-living species from the genera *Prosthecochloris*, *Chlorobium*, and *Chlorobaculum*. However, *Chlorobium chlorochromatii* can form a prokaryotic symbiosis with another bacterium “*Candidatus* Symbiobacter mobilis” in the phototrophic consortium “*Chlorochromatium aggregatum*”^14^. These two species can benefit from each other through metabolic exchange, sensing and moving towards light and sulfide, and electron cycling^15^.

Compared with the well-known coral-algal symbiosis, coral-microbial symbiosis remains largely unknown because limited evidence has been achieved to address this issue. Coral metagenomic studies have been performed previously^16–19^, but metagenomics-based genomic analysis of potential coral microbial symbionts is still a knowledge gap that requires to be filled. Current understanding of potential coral-microbial symbiosis mostly comes from 16S rRNA gene based analyses^20, 21^. The purpose of this study was therefore to explore potential microbial symbionts in corals through massively parallel sequencing of 16S rRNA gene amplicons, to recover potential genomes through metagenomics-based genome binning, and to elucidate potential symbiotic relationships with coral hosts on a genomic scale. In the present study, we enriched microbial cells for coral metagenomic analysis, successfully identified a green sulfur bacterium “*Candidatus* Prosthecochloris korallensis” as a potential microbial symbiont in corals, and analysed the metabolic pathways of the bacterium to gain insights into the mechanisms of coral-microbial symbiosis. This study will provide a new understanding of coral-microbial symbiosis on a genomic scale.

## Results and Discussion

### Discovery of “*Ca*. P. korallensis” in hard corals

**Figure 1:**
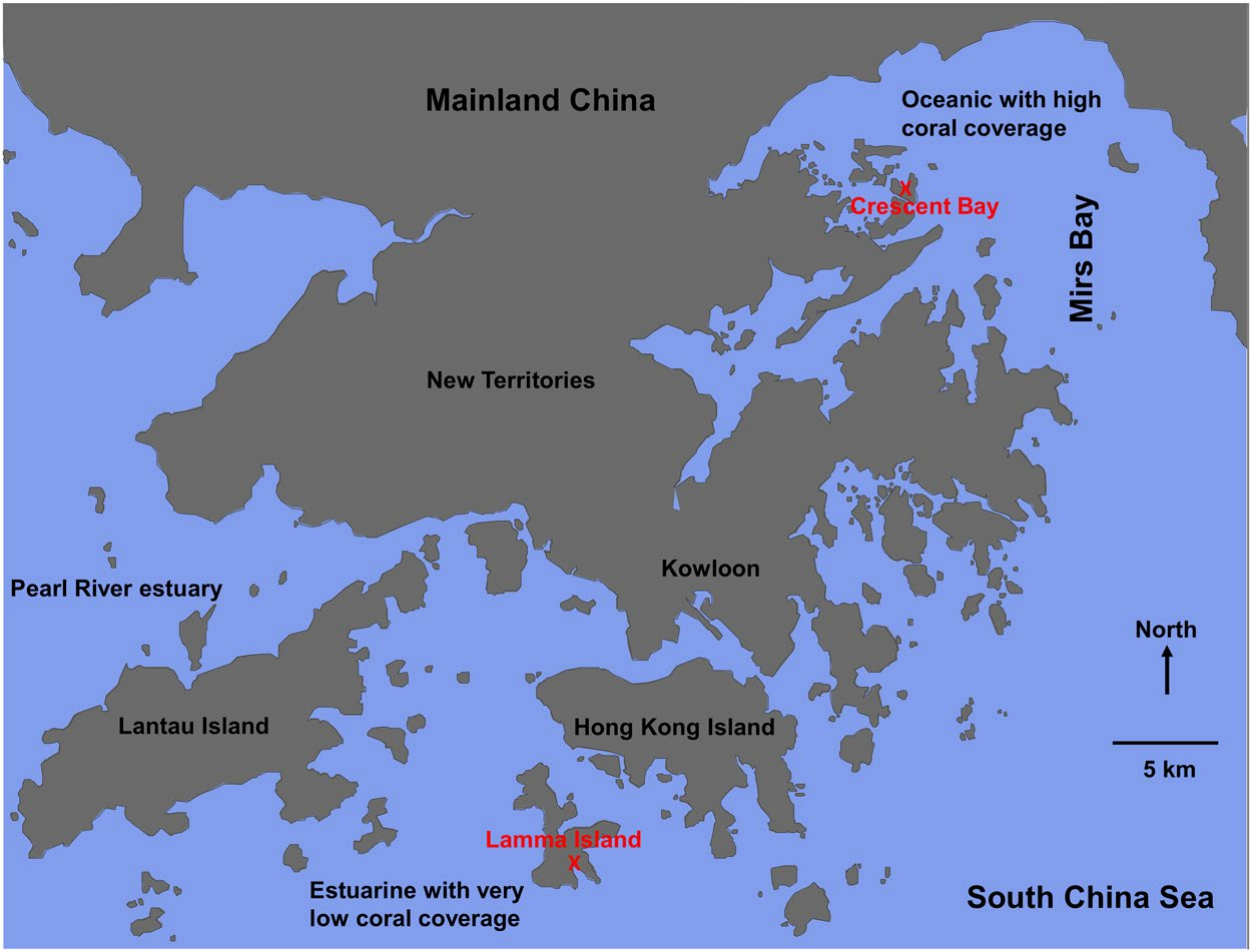
Coral sampling map for the present study. Corals grown in Lamma Island and Crescent Bay were collected in winter (March) and summer (October), which is located in the southwest and northeast of Hong Kong respectively. The geographic map was generated by Ocean Data View 4.7.2 (Schlitzer, R., Ocean Data View, odv.awi.de, 2017).

**Figure 2:**
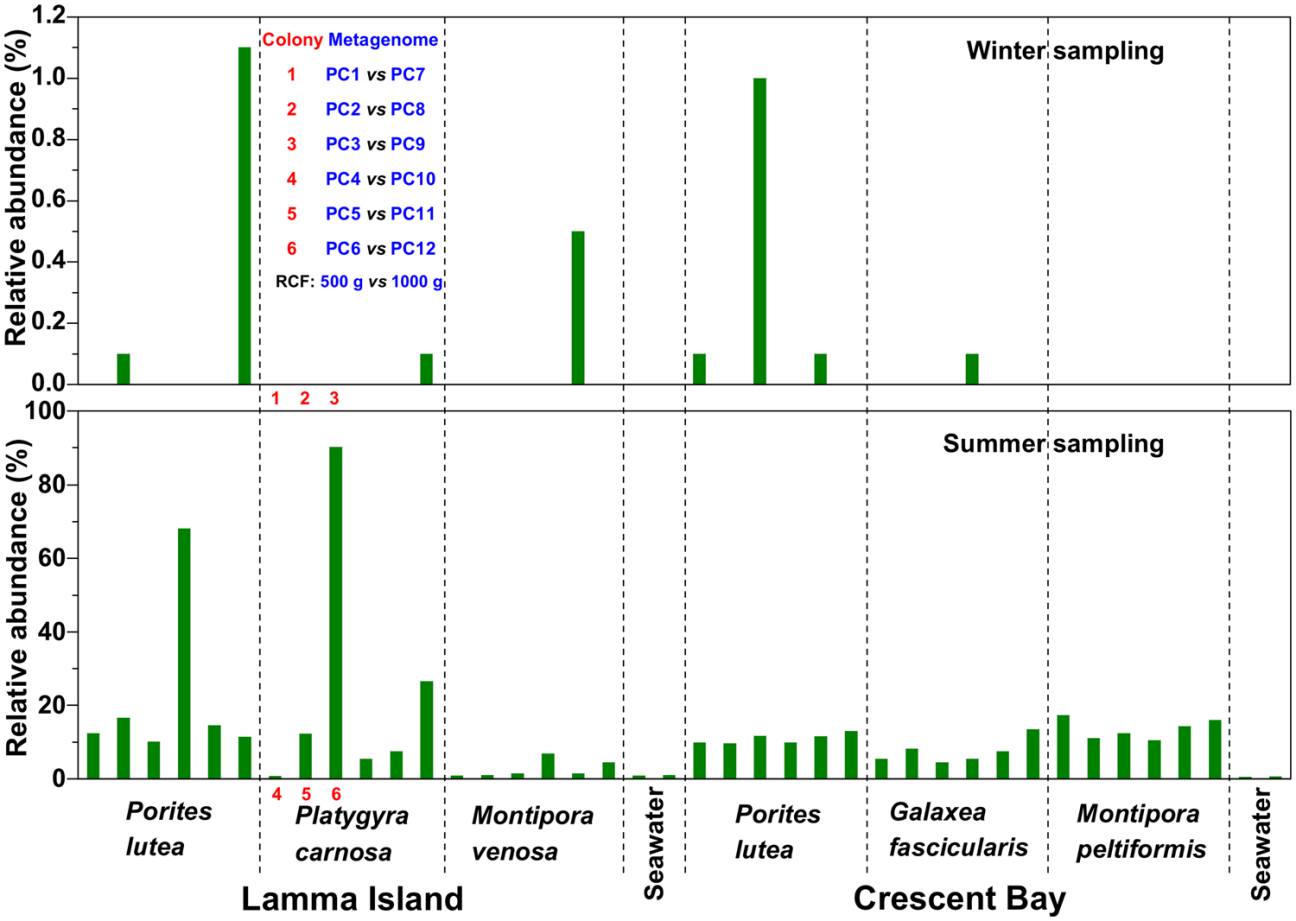
Relative abundance of “*Ca*. P. korallensis” revealed by 16S amplicon sequencing and analysis. The labels showing 1–3 in winter sampling and 4–6 in summer sampling were samples collected from six colonies of *P. carnosa* which were used for metagenomic study to recover the genome of “*Ca*. P. korallensis”. Colonies 1–6 of *P. carnosa* were enriched twice to generate metagenomes PC1–6 (500 g RCF) and PC7–12 (1000 g RCF), respectively. “PC” is the abbreviation of the initial letters of the species name “*Platygyra carnosa*”.

To explore ecologically important bacterial species in local corals, we studied samples of *Porites lutea*, *Platygyra carnosa* and *Montipora venosa* from Lamma Island and *Porites lutea*, *Galaxea fascicularis* and *Montipora peltiformis* from Crescent Bay collected in both winter and summer, 2014 (Fig. 1 and Table S1). As shown in Fig. 2, multiplexed 16S amplicon sequencing identified an operational taxonomic unit (OTU), i.e. “*Ca*. P. korallensis”, which was highly abundant (0.7–90.1%, mean 13.42%) in summer coral samples, but had a very low abundance (~0–1.1%, mean 0.09%) in winter coral samples. For comparison, its relative abundance in seawater samples was 0.5–1% (mean 0.75%) in summer and 0% in winter. This finding indicates that “*Ca*. P. korallensis” propagates well in summer but not well in winter and that it grows well in corals but not well in seawater. Because members of *Prosthecochloris* are green sulfur bacteria and “*Ca*. P. korallensis” can be highly abundant in local corals, “*Ca*. P. korallensis” may play an important role in the coral holobionts. To reveal its potential ecological functions, we have recovered its genome from the coral metagenome. As shown in Fig. 2, coral colonies 1–3 (winter samples) and 4–6 (summer samples) of *P. carnosa* were selected for the following metagenomic study.

### Enrichment effect of “*Ca*. P. korallensis” in coral metagenome

**Figure 3:**
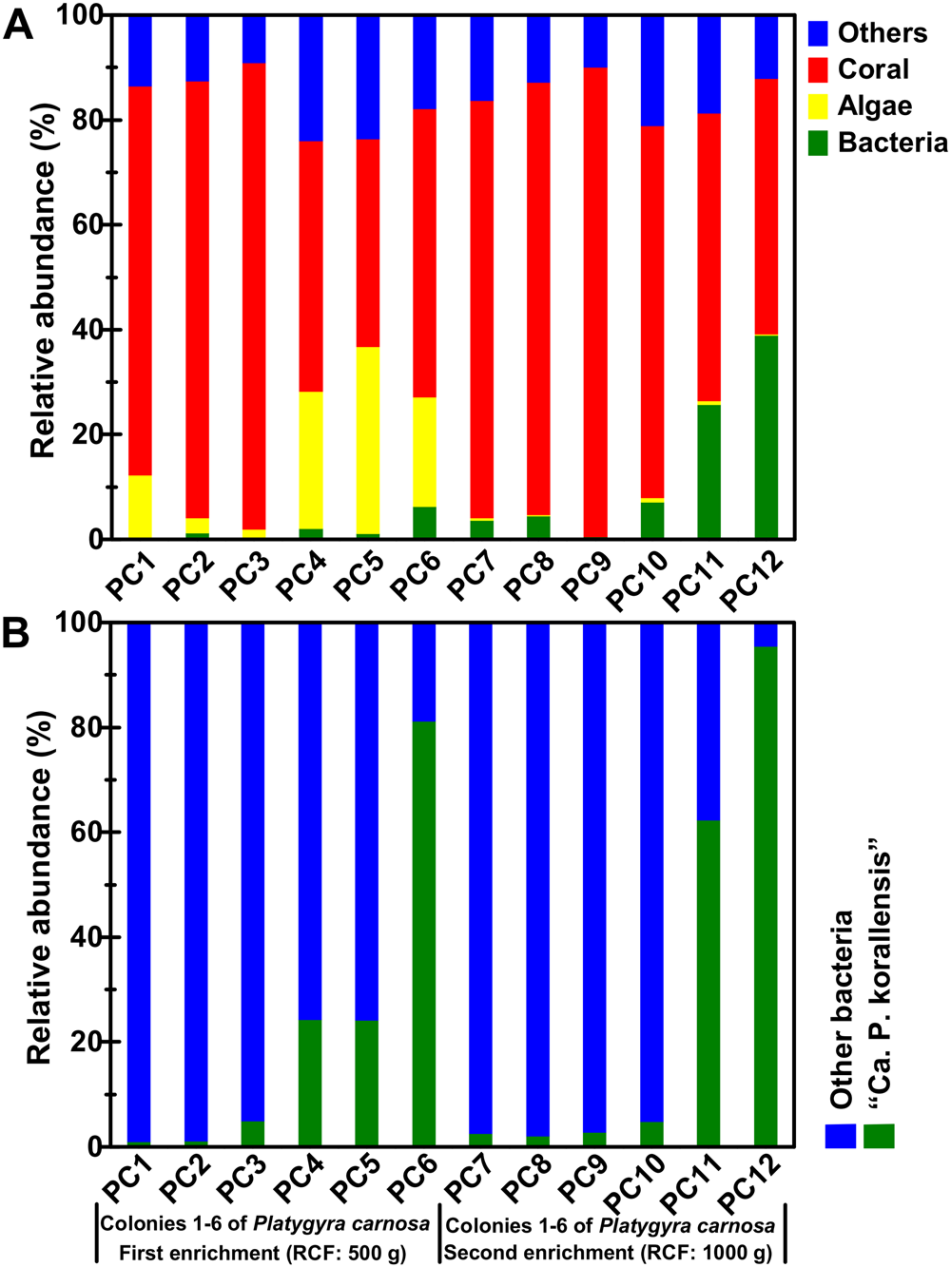
Enrichment effects of “*Ca*. P. korallensis” in coral metagenomic sequencing datasets (**A**) and 16S amplicon sequencing datasets (**B**). PC1–6 and PC7–12 represent the first (500 g RCF) and the second (1000 g RCF) batch of microbial cell enrichment, respectively. “PC” is the abbreviation of the initial letters of the species name “*Platygyra carnosa*”.

Information for the 12 metagenome sequencing datasets before and after quality control is summarized in Table S2. The percentage of clean bases was 90–92% and 2.4–3.5 giga base pairs (Gb) of clean data were finally obtained for each sample. The microbial enrichment effect was evaluated through metagenome sequencing and 16S amplicon sequencing, both of which were conducted for the 12 enriched DNA samples (Fig. 3A). The result showed that the enrichment effect for the second batch (PC7–12) was much better than the first batch (PC1–6) because more bacteria were obtained in the second batch than the first batch. Algae were removed effectively in the second batch, but much more were retained in the first batch. It is important to note however, that coral and other components could not be removed in both batch enrichments. These findings suggested that microbes could be enriched efficiently when algae were removed successfully, and that 500 g relative centrifugal force was insufficient to pellet the algal cells but 1000 g was sufficient. The 16S amplicon sequencing data revealed that the target OTU “*Ca*. P. korallensis” was highly enriched in datasets PC6, PC11, and PC12 (Fig. 3B). This finding was consistent with the result of the metagenome sequencing data (Figure S1). Hence, the PC6, PC11, and PC12 datasets were selected for the following metagenome assembly. “*Ca*. P. korallensis” was absent with no enrichment for colonies 1–3 (Fig. 2) but it was detected with enrichment in PC1–3 and PC 7–9 (Fig. 3B), indicating “*Ca*. P. korallensis” actually was present in winter corals but at a very low level. The presence of “*Ca*. P. korallensis” in winter corals demonstrates that it is not an opportunistic colonist of corals.

### Genome recovery of “*Ca*. P. korallensis” through genome binning

**Figure 4:**
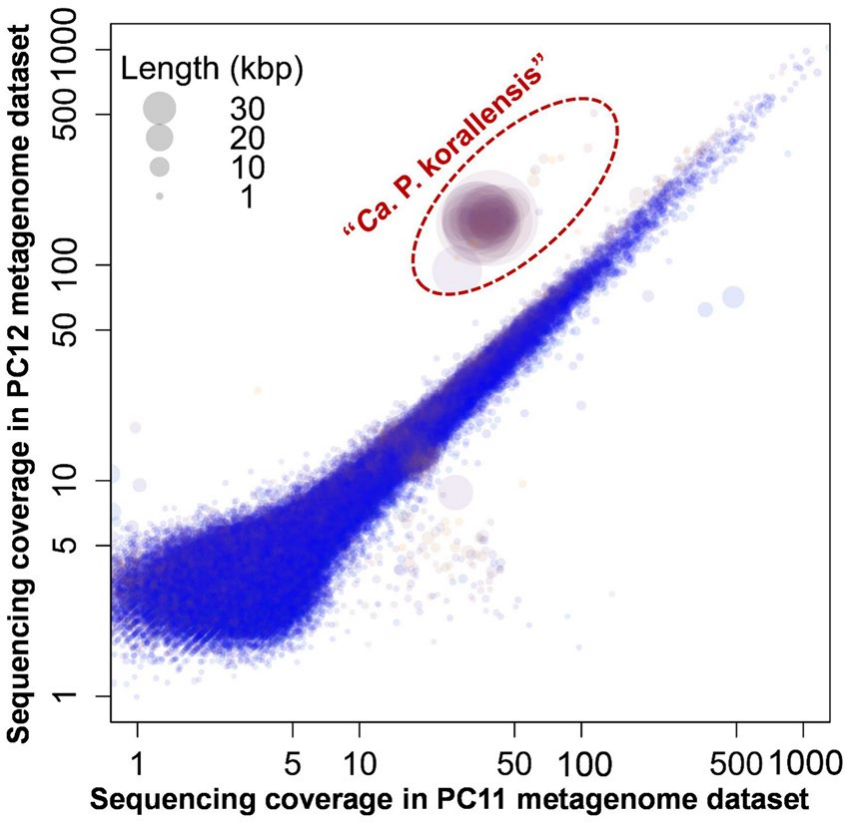
Genome recovery of “*Ca*. P. korallensis” from the coral metagenome datasets PC11 and PC12 using the differential coverage genome binning method.

Metagenome assembly using SPAdes was conducted on the PC6, PC11, and PC12 datasets individually. The best assembly for each dataset was used for the following genome binning. The draft genome of “*Ca*. P. korallensis” (Supplementary Dataset 1) was successfully recovered in PC12 assembly using differential sequencing coverage of PC11 and PC12 metagenome datasets (Fig. 4). There were about 37-fold and 160-fold sequencing coverages for the target genome in the PC11 and PC12 datasets, respectively. Additionally, the draft genome of “*Ca*. P. korallensis” could be recovered from any one assembly of PC6, PC11, and PC12 using any two datasets from them (data not shown). The draft genome of “*Ca*. P. korallensis” consists of 68 contigs, with a size of 2,583,763 base pairs (bp). The longest contig and N50 size is 264,073 bp and 106,492 bp, respectively. The quality of the genome was further assessed with its relatives using two calculations (Table 1). The draft genome of “*Ca*. P. korallensis” was nearly complete and recovered with an extremely low level of contamination, i.e. 99.45% completeness and 1.10% contamination, estimated by CheckM. The draft genome of “*Ca*. P. korallensis” has 2,514 genes, including 2,482 protein-coding genes, 29 tRNA genes, and 3 rRNA genes, and the GC content is 48.3%.

**Table 1:** Genome comparison and quality validation between “*Ca*. P. korallensis” and its relatives. There are eight relatives for which genome is available including *Chlorobium phaeobacteroides* BS1, *Prosthecochloris aestuarii* DSM 271, *Chlorobium limicola* DSM 245, *Chlorobium phaeobacteroides* DSM 266, *Chlorobium ferrooxidans* DSM 13031, *Chlorobium phaeovibrioides* DSM 265, *Chlorobaculum parvum* NCIB 8327, and *Chlorobaculum tepidum* TLS. Total and unique essential single-copy genes (ESCGs) were evaluated using the genome binning program package. Completeness (%) and contamination (%) were validated by CheckM.

Genome	Size (Mb)	GC%	Protein	rRNA	tRNA	Total ESCGs	Unique ESCGs	Completeness	Contamination
“*Ca*. P. korallensis”	2.58	48.3	2,482	3	29	105	104	99.45	1.10
BS1	2.74	48.9	2,588	6	46	106	105	99.45	0.00
DSM 271	2.58	50.1	2,379	3	46	106	104	98.90	0.27
DSM 245	2.76	51.3	2,560	6	48	106	105	99.98	0.00
DSM 266	3.13	48.4	2,881	6	47	106	105	99.45	0.00
DSM 13031	2.54	50.1	2,338	3	46	91	90	95.66	0.00
DSM 265	1.97	53.0	1,793	3	45	108	106	98.36	1.09
NCIB 8327	2.29	55.8	2,098	6	51	107	105	98.89	0.28
TLS	2.15	56.5	2,008	6	50	106	105	98.90	0.55

### Phylogenetic analysis between “*Ca*. P. korallensis” and its relatives

**Figure 5:**
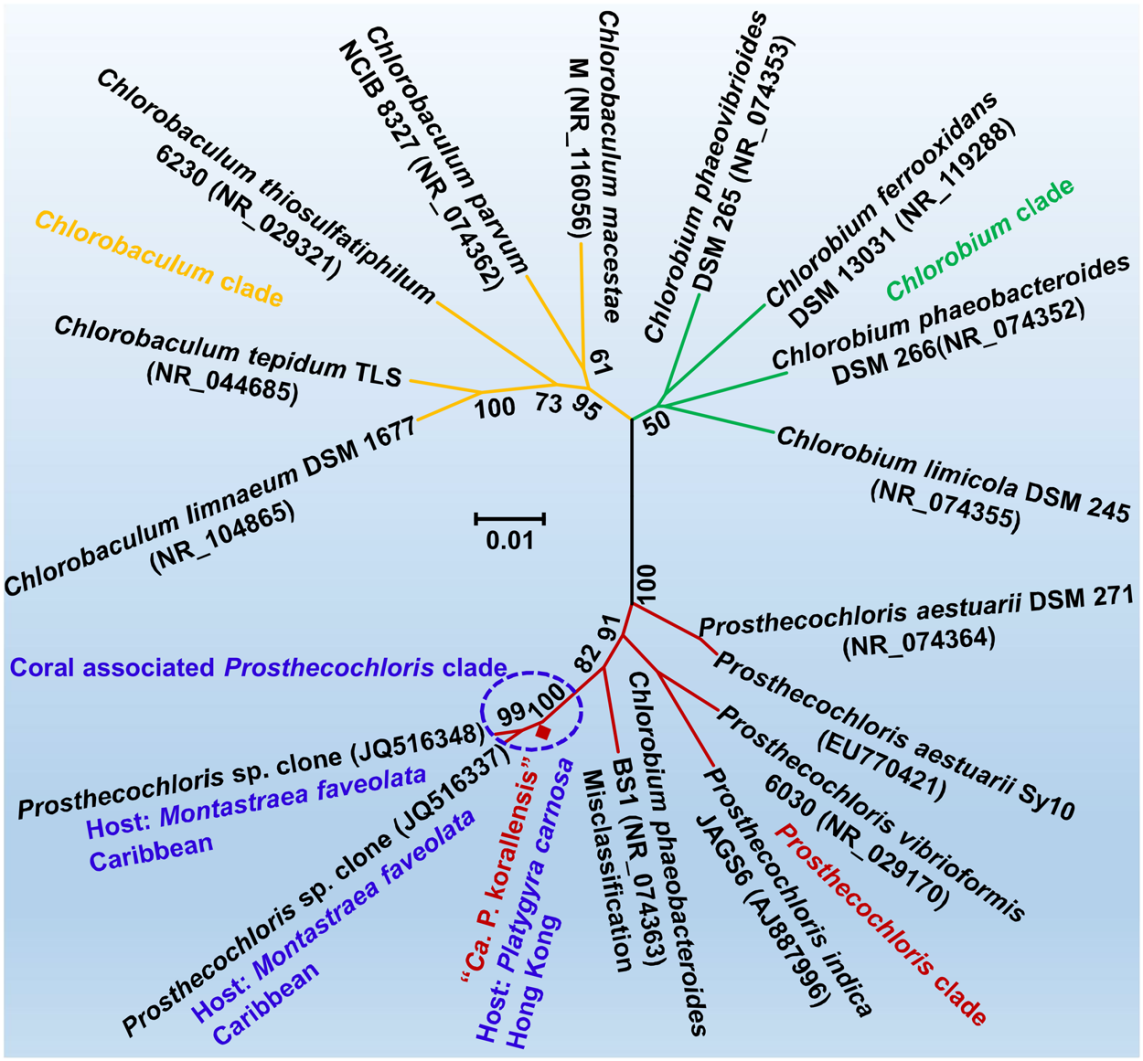
Phylogenetic analysis for “*Ca*. P. korallensis” and its relatives using full-length 16S rRNA gene. The neighbour-joining tree was constructed using MEGA 6.06 with 1000 bootstrap replication test. The bootstrap values higher than 50% are shown. The scale bar 0.01 indicates 1% nucleotide substitution.

The unrooted full-length 16S phylogenetic tree illustrates the evolutionary relationships between “*Ca*. P. korallensis” and its relatives (Fig. 5). The *Prosthecochloris*, *Chlorobium*, and *Chlorobaculum* clades are clearly divided, representing the most common genera in the phylum *Chlorobi*. These genera belong to the green sulfur bacterial family *Chlorobiaceae*. Interestingly, “*Ca*. P. korallensis” and two *Prosthecochloris* clones derived from the Caribbean coral *Montastraea faveolata* are closely clustered into a coral-associated *Prosthecochloris* clade, sharing ~99.5% identity in nearly full-length of the 16S rRNA gene sequence^22^. The other *Prosthecochloris* species are free-living isolates, which are phylogenetically distant from the coral-associated *Prosthecochloris* clade. This suggests that “*Ca*. P. korallensis” might live a different lifestyle. The phylogenetic analysis classified the species into the genus *Prosthecochloris*. We tentatively propose the name “*Candidatus* Prosthecochloris korallensis”, where korallensis is a latinized form of koráli, the Greek word for coral, reflecting it was found in corals.

### Genome comparison between “*Ca*. P. korallensis” and its relatives

**Figure 6:**
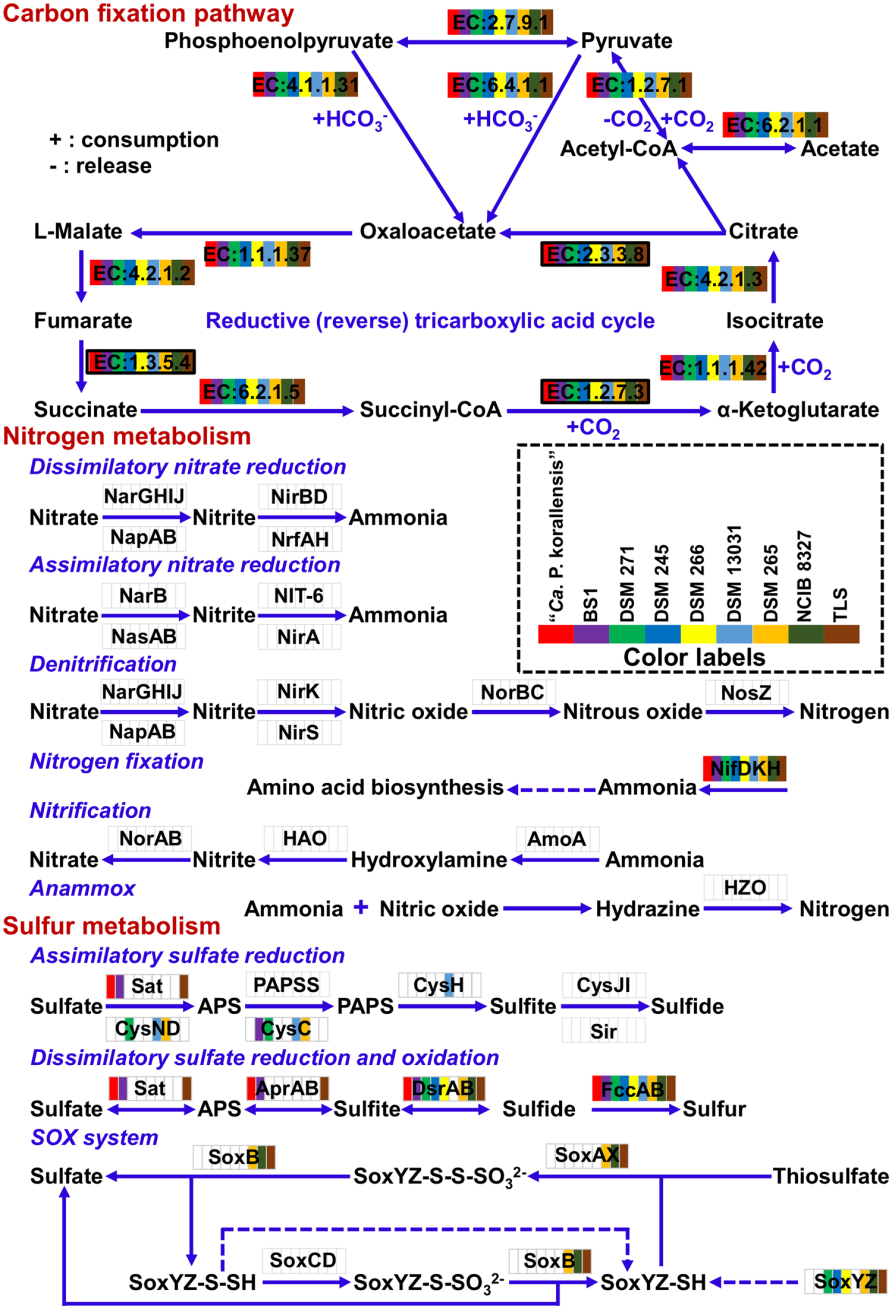
Carbon, sulfur, and nitrogen metabolism for “*Ca*. P. korallensis” and its relatives. Each color indicates a specific bacterium with a certain function. Blank shows lack of a certain function. Black boxes represent three key enzymes for rTCA cycle including ATP citrate lyase, fumarate reductase, and ketoglutarate synthase. All compared genomes have a complete carbon fixation pathway via rTCA cycle, but nitrogen fixation is the only pathway for inorganic nitrogen metabolism. All the genomes compared exhibit the abilities to oxidize sulfide to sulfur, but several of them might even oxidize sulfide to sulfate including “*Ca*. P. korallensis”, BS1, and TLS. “*Ca*. P. korallensis” has no ability to convert thiosulfate to sulfate through the thiosulfate-oxidizing SOX enzyme system.

The genome properties of “*Ca*. P. korallensis” and eight relatives for which genome is available are summarized in Table 1. The draft genome of “*Ca*. P. korallensis” has a size of 2.58 mega base pairs (Mb), which is in the middle of the genome size range (1.97–3.13 Mb) for the nine species. However, “*Ca*. P. korallensis” has the lowest GC content and the smallest number of tRNA genes. Each genome has 1–2 copies of the ribosomal RNA operon. All the genomes compared have a complete carbon fixation pathway via the rTCA cycle (Fig. 6). Each cycle fixes two molecules of carbon dioxide and produces one molecule of acetyl-CoA, which is then assimilated into pyruvate and phosphoenolpyruvate to form central metabolites and to synthesize cellular components. Most of the enzymes involved in the rTCA cycle are shared with the TCA cycle, but three key enzymes, i.e. ATP citrate lyase, fumarate reductase, and ketoglutarate synthase (labelled in Fig. 6), allow the TCA cycle to run in the reverse direction^11^. Nitrogen fixation is the only inorganic nitrogen metabolism pathway found in all of the compared genomes (Fig. 6). This pathway allows these bacteria to convert environmental nitrogen gas into nitrogenous nutrients. Although the genomes compared share the same carbon and nitrogen fixation pathways, they show different sulfur metabolism capabilities (Fig. 6). For example, “*Ca*. P. korallensis” might oxidize sulfide to sulfate to produce more electrons for carbon reduction in the rTCA cycle, but it is unable to convert thiosulfate to sulfate through the thiosulfate-oxidizing SOX enzyme system. All genomes compared have a complete pathway for bacteriochlorophyll (BChl) biosynthesis (Figure S2), which is essential for the development of photosynthetic apparatus in green sulfur bacteria, i.e. chlorosomes. These results show that “*Ca*. P. korallensis” has many genomic features in common with its relatives.

### Core metabolic pathways of “*Ca*. P. korallensis”

**Figure 7:**
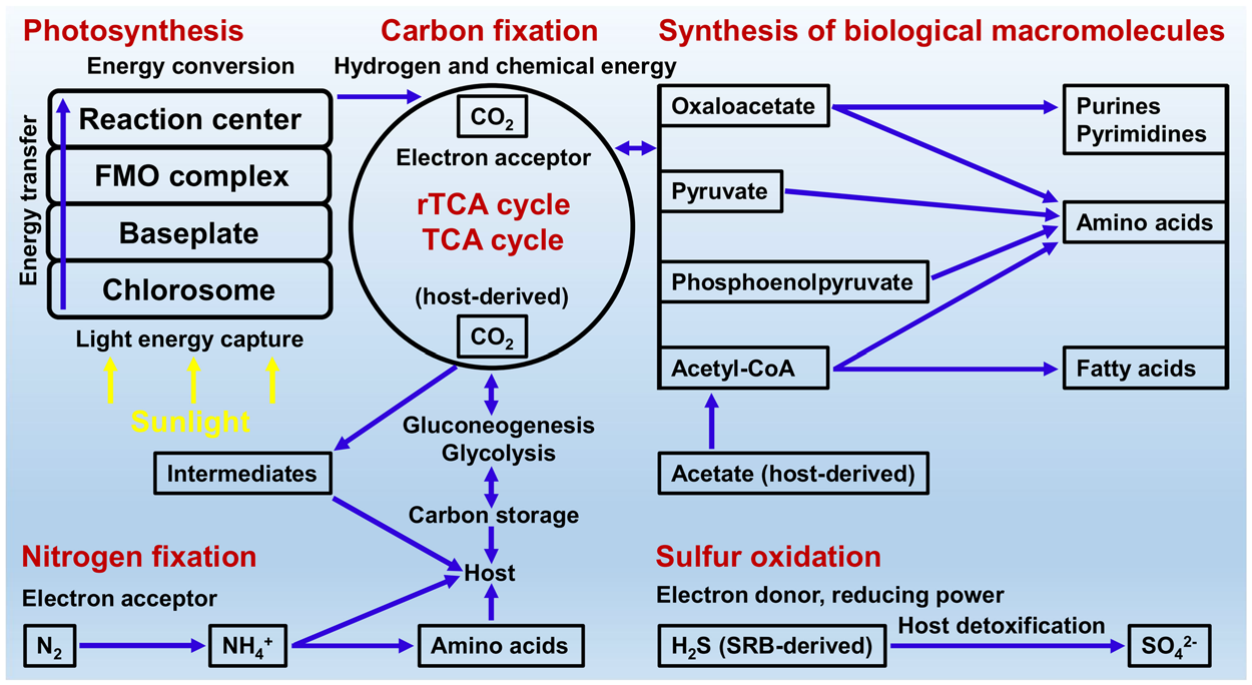
Global metabolic pathways for “*Ca*. P. korallensis” and potential host-microbial interactions. Light energy is harvested and converted into chemical energy, which is used to assimilate carbon dioxide in the rTCA cycle. The intermediates generated in the rTCA cycle might be used for the biosynthesis of fatty acids, amino acids, purines, and pyrimidines, which are essential for the synthesis of biological macromolecules. The fixed carbon and nitrogen sources by “*Ca*. P. korallensis” might be served as host’s nutrients. Host-derived carbon dioxide and acetate and host SRB-derived hydrogen sulfide might be vital for the survival of “*Ca*. P. korallensis”.

A schematic representation based on genomic analysis is proposed to provide a better understanding of how “*Ca*. P. korallensis” captures sunlight and converts it to chemical energy and how “*Ca*. P. korallensis” fixes carbon dioxide and converts it to cell components (Fig. 7). Light energy is captured by the light-harvesting pigments (e.g. BChl *c*, *d*, or *e*) in the photosynthetic apparatus over a broad spectrum. The light energy is then transferred to the baseplate (i.e. the complex of chlorosome protein CsmA and BChl *a*), to the Fenna-Matthews-Olson complex (i.e. the complex of protein and BChl *a*), and finally to the cytoplasmic membrane bound reaction centre (i.e. the complex of proteins, BChl *a*, and other co-factors)^23^. Hydrogen and chemical energy generated in the reaction centre are used to assimilate carbon dioxide in the rTCA cycle. The intermediate products of the rTCA cycle such as acetyl-CoA, pyruvate, phosphoenolpyruvate, and oxaloacetate might be used in the biosynthesis of fatty acids, amino acids, purines, and pyrimidines, which are essential for the further synthesis of biological macromolecules. The assimilated carbon dioxide and chemical energy might be stored in a stable form via gluconeogenesis using phosphoenolpyruvate as the substrate (Figure S3). Potential glycolysis (Figure S3) and TCA cycle (Figure S4) pathways are found in the genomes of “*Ca*. P. korallensis” and its relatives, indicating that “*Ca*. P. korallensis” may utilize carbon storage for cell activities when needed. It has been experimentally demonstrated that the TCA cycle also works in a green sulfur bacterium^11^. Genomic and experimental analyses reveal that green sulfur bacteria are able to assimilate a few organic compounds like acetate and pyruvate, but acetate or pyruvate cannot be used as the sole carbon source for growth without carbon dioxide^11, 24^. Thus, carbon dioxide is not only required for the rTCA cycle but is essential in the assimilation of acetate and pyruvate (Fig. 6). Therefore, “*Ca*. P. korallensis” grows photoautotrophically when it uses carbon dioxide only, but it might grow mixotrophically when additional acetate or pyruvate is available.

### Potential interactions between “*Ca*. P. korallensis” and coral host

A host-microbial interaction profile using the genomic information of “*Ca*. P. korallensis” and the current understanding of green sulfur bacteria is summarized in Fig. 7. The coral host might provide an anaerobic environment in coral tissue or/and skeleton which is essential for “*Ca*. P. korallensis” survival and anoxygenic photosynthesis. Carbon dioxide produced by host and hydrogen sulfide generated by host sulfate-reducing bacteria (SRB) might serve as the carbon source and electron donor for the photoautotrophic growth of “*Ca*. P. korallensis”, both of which might be contributed by the coral host. It has been demonstrated that the growth of green sulfur bacterium *C. tepidum* TLS enhanced significantly with the addition of acetate, and its genome is also compared in this study^11^. In addition, the coral host can produce abundant acetate for *Symbiodinium* to perform lipid synthesis for it^3^, which is a possible acetate source for “*Ca*. P. korallensis”. According to these evidences, host-derived acetate might stimulate the multiplication of “*Ca*. P. korallensis” through acetate assimilation pathway in the mixotrophic growth, which might happen in the summer bloom period. Hydrogen sulfide is very toxic to many marine invertebrates by inhibiting cytochrome c oxidase and a type of catalase^25, 26^. “*Ca*. P. korallensis” might help coral host detoxify sulfide through oxidation of sulfide to sulfate. “*Ca*. P. korallensis” might also provide fixed carbon and nitrogen, intermediates of the rTCA/TCA cycles, sugars, and other nutrients to the coral host. These potential interactions between “*Ca*. P. korallensis” and its host suggest it might be a coral symbiont. However, follow-up experiments would be needed to truly confirm that this bacterium is a symbiont providing some sort of benefit to the coral.

### Distribution of “*Ca*. P. korallensis” in coral host

A recent study reported that *Prosthecochloris* was dominant in the microbiome of the green layer in the skeleton beneath coral tissue^27^, but the tissue and surrounding seawater microbiomes were not investigated. Another study examined seasonal changes in coral mucus, tissue, skeleton, and seawater microbiomes, and the results revealed that *Prosthecochloris* was much more abundant in tissue and skeleton than in mucus, but that it was rare in winter samples and nearly absent in the seawater^28^. This observation is consistent with the present study (Fig. 2), which employed the composite tissue and surface skeleton for experiments. Unfortunately, the previous and present studies did not specifically localize “*Ca*. P. korallensis” in coral tissue or/and skeleton. But the genomic evidence of “*Ca*. P. korallensis” suggests it lives in the holobionts where light can reach and an anaerobic environment can form. This is understandable because the highly efficient photosynthesis using the unique light harvesting organelles of green sulfur bacteria allows them to inhabit the only lowermost part of stratified environments where minimum light intensity is available^29^. It is also consistent with “*Ca*. P. korallensis” being nearly absent in seawater because of its oxygen sensitivity and reliance on the coral host. The significantly decreased population of “*Ca*. P. korallensis” in winter compared with summer may be primarily caused by a low level of light intensity and temperature in winter and insufficient hydrogen sulfide because sulfate-reducing activity in the sea is controlled by temperature and organic loading, with nearly an order of magnitude difference between summer and winter^30^.

### Prevalence of green sulfur bacteria in hard corals

Previous 16S rRNA gene based studies revealed the presence of potential green sulfur bacteria in the phylum *Chlorobi* with varying abundances in the hard coral *Isopora palifera* from southern Taiwan^27, 31^, *P. lutea* from the South China Sea^28, 32, 33^, *Turbinaria mesenterina* from subtropical Australia^34^, *Porites astreoides* from southern Caribbean^35^, and *Mussismilia* spp. from the southern Atlantic^36^. In addition, they were also found in the Antarctic soft coral *Alcyonium antarcticum* ^37^, ascidians of the Great Barrier Reef^38^, and the deep lake water sponge *Baikalospongia intermedia* ^39^. These findings suggest that green sulfur bacteria are prevalent in aquatic animals, and might play an important role in the host. A clone library based study revealed that the dominant nitrogen-fixing bacteria were *Chlorobi* and *Cyanobacteria* in healthy and diseased *P. lutea*, respectively^33^. *Chlorobi* was very dominant in apparently healthy colonies of *I. palifera* ^27^ and *P. lutea* ^28^, and was also predominant in healthy colonies of five coral species in this study (Fig. 2). These observations suggest that abundant green sulfur bacteria do not cause damage to the coral host under the conditions tested.

## Conclusions

In the present study, we discovered an ecologically important bacterial species “*Ca*. P. korallensis” through 16S massively parallel sequencing and analysis. We enriched microbial cells in corals for metagenomic analysis and recovered its genome with high-quality through metagenomics-based genome binning. Genomic analysis reveals it is a green sulfur bacterium with potentially important ecological functions such as anoxygenic photosynthesis, carbon fixation via the rTCA cycle, nitrogen fixation, and sulfur oxidization. Host-microbial interaction reveals a potential symbiotic relationship and suggests that it might be a coral symbiont. The present study provides genomic insights into a potential microbial symbiont in corals and achieves a novel understanding of coral-microbial symbiosis on a genomic scale. Moreover, the success of recovery of microbial genome from coral metagenome here exhibits technical importance for future coral metagenomic study.

## Methods

### Coral sampling

Hong Kong is located in the northern border of the South China Sea and provides a subtropical habitat for coral development. Lamma Island (Western Hong Kong) is an estuarine environment grown with a very low coverage of coral due to the impact of freshwater runoff and pollution from the Pearl River. While Crescent Bay (Eastern Hong Kong) is an oceanic environment for coral development and thus the coral coverage is high. The two locations with contrasting environments were selected for collection of coral and seawater samples (Fig. 1). Additionally, both winter and summer samples were taken providing seasonal comparison. According to the seawater temperature monitoring data, corals collected in March and October were employed to represent winter and summer samples, respectively. In total, five coral species were used in this study: *Platygyra carnosa*, *Porites lutea*, *Montipora venosa*, *Montipora peltiformis*, and *Galaxea fascicularis*. Small pieces of coral skeleton with tissue were collected from apparently healthy colonies and packaged into pre-tagged sterile zipper bags. Six colonies representing biological replicates were collected for each coral species and corresponding seawater samples were also collected to determine the relationship between microbes in the environment and those associated with coral. Information about the samplings is detailed in Table S1. After sampling, the collected corals were immediately washed with sterilized seawater to remove microbes loosely attached to the coral surface, then fixed in 70% ethanol and kept on dry ice. The collected seawater was filtered through a 0.22-μm polycarbonate membrane to concentrate seawater microbes. All fixed samples were kept at −30 °C until DNA extraction.

### Species and colonies selected for coral metagenomic study through 16S amplicon sequencing and analysis

Due to the large number of coral samples (72 in total), it was necessary to conduct 16S amplicon sequencing and analysis to target certain samples for metagenomic study. The total DNA of all collected coral and seawater samples was extracted to serve as PCR templates. The 16S V3-V4 regions were amplified using barcoded primer sets 341F (5′-CCTAYGGGRBGCASCAG-3′) & 802R (5′-TACNVGGGTATCTAATCC-3′)^40^ and sequenced massively using an Illumina MiSeq sequencer^41, 42^. The 16S amplicon sequencing datasets have been deposited in the NCBI Sequence Read Archive under accession number SRP066229. The 16S data were cleaned and analysed using QIIME^43^. The 97% operational taxonomic units (OTUs) were picked and profiled to identify microbes potentially correlated to corals. An interesting microbe “*Ca*. P. korallensis” was discovered to be potentially associated with the investigated corals we collected for the present study. To reveal its ecological roles in corals, we tried to recover its genome through metagenomics-based genome binning. Here, we selected *P. carnosa* as the target species and employed three summer colonies and three winter colonies for the following metagenomic study based on the considerations: i) it is one of the dominant species in Hong Kong and its population has been affected by various threats^44^; ii) one of the selected colonies harboured the highest relative abundance of “*Ca*. P. korallensis”, as high as 90.1%; iii) the selected three summer colonies formed the best gradients for the relative abundance of “*Ca*. P. korallensis”, which is essential for genome binning; iv) the selected three winter colonies all showed absence of “*Ca*. P. korallensis”, which is critical to further confirm whether it exists in winter corals or not.

### Coral microbial cell enrichment, metagenomic DNA extraction, and metagenome sequencing

Coral metagenomic study is challenging because it is difficult to enrich associated microbes in the coral holobiont. We followed the well-established method^19^ to develop a simple enrichment protocol. Six selected colonies of *P. carnosa* were used for metagenomic study including colonies 1–3 collected in winter and colonies 4–6 collected in summer (Fig. 2). Each coral sample (~2 cm × 2 cm) was firstly rinsed with 1 × PBSE (137 mM NaCl, 2.7 mM KCl, 4.3 mM Na_2_HPO_4_·7H_2_O, 1.4 mM KH_2_PO_4_ and 10 mM EDTA) and fully ground in 1 × PBSE using mortar and pestle. The resultant coral slurry was transferred into a 50 ml falcon tube for 5 min vortex and 1 min natural sedimentation to remove large particles. The supernatant was homogenized at 15,000 rpm for 5 min. The homogeneous matrix was centrifuged at 500/1000 g (designed as the first/second batch enrichment) for 15 min under 4 °C to remove algal cells, coral and other large components. The supernatant containing microbial cells was further centrifuged at 15,000 g for 15 min under 4 °C to concentrate microbial cells. The microbial cell pellets were finally used for metagenomic DNA extraction using FastDNA® Spin Kit for Soil (MP Biomedicals, France). The only difference between the two batches of enrichment was the relative centrifugal force (RCF) in the removal of *Symbiodinium*, i.e. 500 g^19^ versus 1000 g^18^. The first and second batch DNA extracts for colonies 1–6 of *P. carnosa* were designated as PC1–6 (500 g RCF) and PC7–12 (1000 g RCF), respectively. “PC” is the abbreviation of the initial letters of the species name “*Platygyra carnosa*”. The extracted DNA samples PC1–12 were sent to Novogene (Beijing, China) for shotgun sequencing on an Illumina HiSeq platform with a paired-end (PE) 125 bp × 2 mode. The obtained metagenomic datasets have been deposited in the NCBI Sequence Read Archive under accession number SRP066452. Additionally, the 12 metagenomic DNA extracts were further analysed by 16S amplicon sequencing, as described above.

### Data filtration and evaluation of microbial cell enrichment effect

The raw metagenome sequences were trimmed to remove low-quality reads using the NGS (next-generation sequencing) QC (quality control) Toolkit^45^ using default settings. The generated 12 clean metagenomic datasets (Table S2) were searched against the Silva SSU (small subunit) rRNA gene database^46^ using BLASTN with an e-value of 1 × 10^−20^ to recover the best hits. The SSU rRNA gene hitting reads were extracted and re-searched against the NCBI nt database using the same e-value to output 50 hits for each search. The BLASTN outputs were imported into MEGAN5^47^ to perform taxonomic annotation and comparison using the LCA (lowest common ancestor) algorithm. Metagenomic analysis using 16S/18S as the fingerprint was combined with 16S amplicon sequencing analysis on PC1–12, which was conducted to evaluate the microbial cell enrichment effect. The metagenomic datasets showing the best performance of microbial cell enrichment were used for metagenome assembly and genome binning.

### Metagenome assembly and genome binning

The selected metagenomic datasets were individually assembled into contigs by SPAdes Genome Assembler 3.1.0^48^ and obtained the best assembly using k-mer size optimization. The draft genome of interest was recovered by a differential coverage genome binning method^49^. Bowtie 2 2.2.3^50^ was employed to map short reads of the metagenomic datasets onto the assembled contigs. The contig sequencing coverage was calculated using SAMtools 0.1.19^51^. The contig GC content and tetranucleotide frequency (TNF) were analysed using the publically available scripts (http://www.nature.com/nbt/journal/v31/n6/full/nbt.2579.html). Prodigal 2.60^52^ was used to predict protein-coding sequences, which were searched against hidden Markov models covering 107 bacterial proteins to identify essential single-copy genes^49^. The taxa of essential single-copy genes were identified by BLASTP against the NCBI nr database and MEGAN5^47^. Genome binning was conducted in the RStudio platform with the scripts developed by Albertsen and his colleagues. All contigs were grouped based on the sequencing coverage, and the group of contigs covering the bacterium of interest was selected for further post-binning refinement using principal component analysis of the TNF. The number of total and unique essential single-copy genes of the draft genome was analysed to evaluate the completeness and contamination. CheckM 1.0.6^53^ was further employed to assess the quality of the recovered draft genome.

### Phylogenetic analysis and taxonomic classification

The ribosomal RNA genes in the binned draft genome were identified by a tool based on hidden Markov models^54^. The 16S rRNA gene sequence was searched against the NCBI nt and 16S rRNA gene databases to collect reference sequences for phylogenetic analysis. The neighbour-joining tree was constructed using MEGA 6.06^55^ with a 1000 bootstrap replication test. Clades were grouped at genus level in the phylogenetic tree and a potential new species was inferred based on the phylogenetic distance with the known species.

### Genome comparison and functional analysis

All available genomes of green sulfur bacteria were downloaded from the NCBI genome database, including *Chlorobium phaeobacteroides* BS1 (NC_010831), *Prosthecochloris aestuarii* DSM 271 (NC_011059), *Chlorobium limicola* DSM 245 (NC_010803), *Chlorobium phaeobacteroides* DSM 266 (NC_008639), *Chlorobium ferrooxidans* DSM 13031 (NZ_AASE00000000), *Chlorobium phaeovibrioides* DSM 265 (NC_009337), *Chlorobaculum parvum* NCIB 8327 (NC_011027), and *Chlorobaculum tepidum* TLS (NC_002932). These genomes were analysed and compared with the draft genome of “*Ca*. P. korallensis”. Prokka 1.10^56^ was employed to annotate all compared genomes individually. The annotated protein sequences of each genome were searched against the online KEGG Automatic Annotation Server^57^. The generated 9 outputs with the KEGG orthology (KO) identifiers (i.e. K numbers) were merged together for metabolic pathway reconstruction and comparison using KEGG Mapper (http://www.genome.jp/kegg/mapper.html).

## Electronic supplementary material

Dataset 1

Supplementary Information
