# Supplementary material for: Metagenomic analysis reveals a green sulfur bacterium as a potential coral symbiont
Source: Sci Rep. 2017 Aug 24;7:9320. doi: 10.1038/s41598-017-09032-4 (PMC5571212; doi:10.1038/s41598-017-09032-4)
Supplement: Supplementary file 2 — Supplementary Information [file 41598_2017_9032_MOESM2_ESM.pdf]

Supplementary Information

Metagenomic analysis reveals a green sulfur bacterium as a potential coral symbiont

Lin Cai[1,#], Guowei Zhou[1,2,#], Ren-Mao Tian[1], Haoya Tong[1], Weipeng Zhang[1], Jin Sun[1], Wei Ding[1], Yue Him Wong[1], James Y. Xie[3], Jian-Wen Qiu[3], Sheng Liu[2], Hui Huang[2,*], Pei-Yuan Qian[1,*]

[1] Shenzhen Research Institute and Division of Life Science, The Hong Kong University of Science and Technology, Hong Kong SAR, China

[2] Key Laboratory of Tropical Marine Bio-resources and Ecology, South China Sea Institute of Oceanology, Chinese Academy of Sciences, Guangzhou, China

[3] Department of Biology, Hong Kong Baptist University, Hong Kong SAR, China

Running title:

Genomic insights into a potential coral symbiont

[#]Equal contribution to this study

[*]**Corresponding author:**

Hui Huang, PhD, Senior Scientist
South China Sea Institute of Oceanology, Chinese Academy of Sciences, 164 West Xingang Road, Guangzhou, China
Phone & Fax: +862-8446-0294 E-mail: huanghui@scsio.ac.cn

Pei-Yuan Qian, PhD, Chair Professor
Division of Life Science, The Hong Kong University of Science and Technology, Clear Water Bay, Hong Kong SAR, China
Tel: +852-2358-7331 Fax: +852-2358-1559 E-mail: boqianpy@ust.hk

List of contents:

Figure S1 Taxonomic comparison of coral metagenome datasets PC1-12 using SSU rRNA gene as the fingerprint. BLASTN was searched against NCBI nt database using Silva SSU rRNA gene database hitting reads. The outputs were imported into MEGAN5 for visualization using the LCA algorithm. Most assignments of Chlorobi, Dinophyceae, and Metazoa covered "*Ca.* P. korallensis", *Symbiodinium*, and *P. carnosa*, respectively.

Figure S2 Bacteriochlorophyll biosynthesis pathway for "*Ca.* P. korallensis" and its relatives. Each color indicates a specific bacterium with certain function. Blank shows lack of certain function.

Figure S3 Gluconeogenesis and glycolysis pathway for "*Ca.* P. korallensis" and its relatives. Each color indicates a specific bacterium with certain function. Blank shows lack of certain function.

Figure S4 TCA cycle for "*Ca.* P. korallensis" and its relatives. Each color indicates a specific bacterium with certain function. Blank shows lack of certain function.

Table S1 Information of sampling locations, coordinates, dates, and species. Six colonies of each species were collected in each sampling.

Table S2 Information of metagenome sequencing datasets before and after quality control. "-1" and "-2" indicate the paired-end read 1 and 2, respectively.

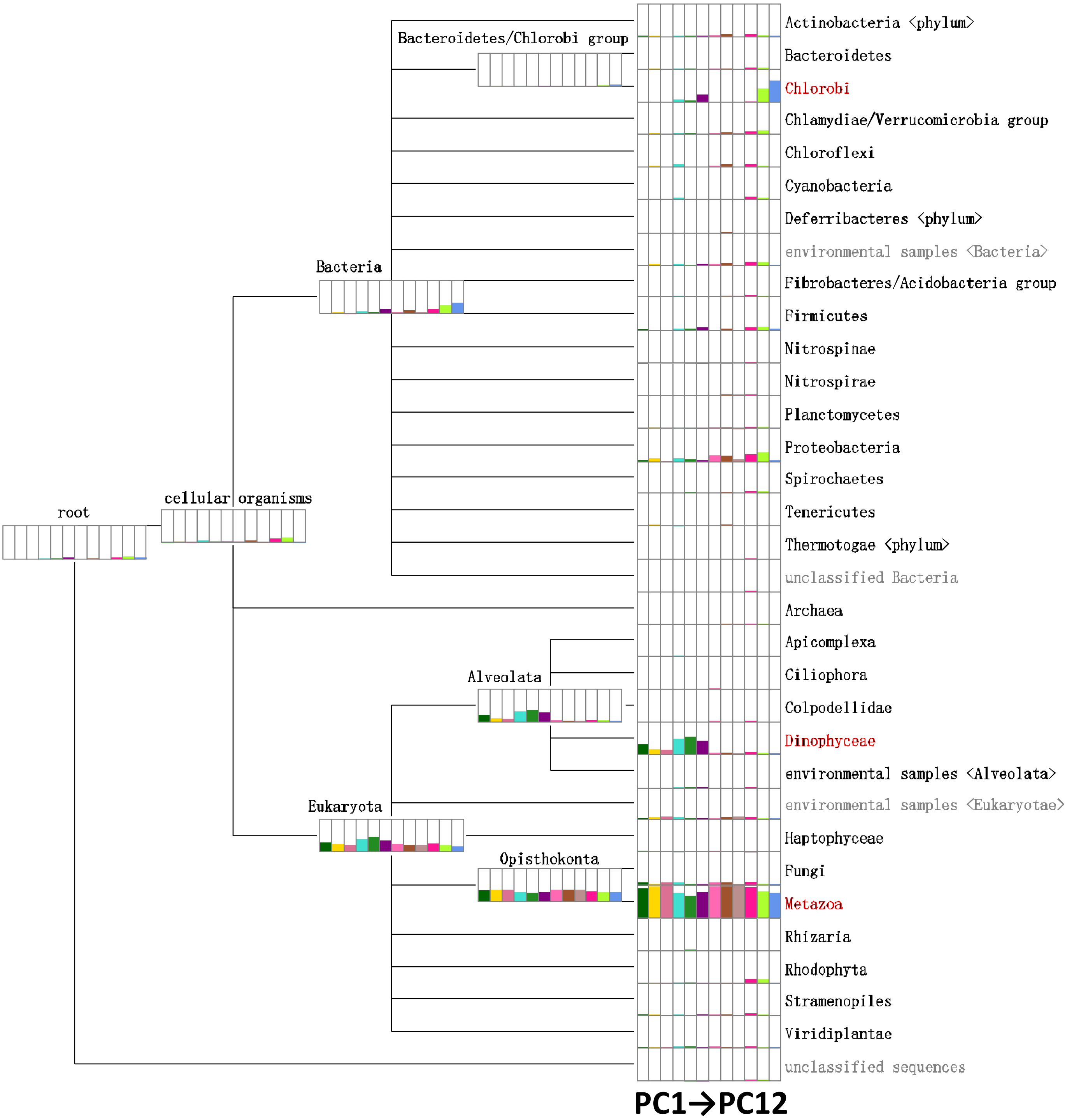

Figure S1 Taxonomic comparison of coral metagenome datasets PC1-12 using SSU rRNA gene as the fingerprint. BLASTN was searched against NCBI nt database using Silva SSU rRNA gene database hitting reads. The outputs were imported into MEGAN5 for visualization using the LCA algorithm. Most assignments of Chlorobi, Dinophyceae, and Metazoa covered "*Ca.* P. korallensis", *Symbiodinium*, and *P. carnosa*, respectively.

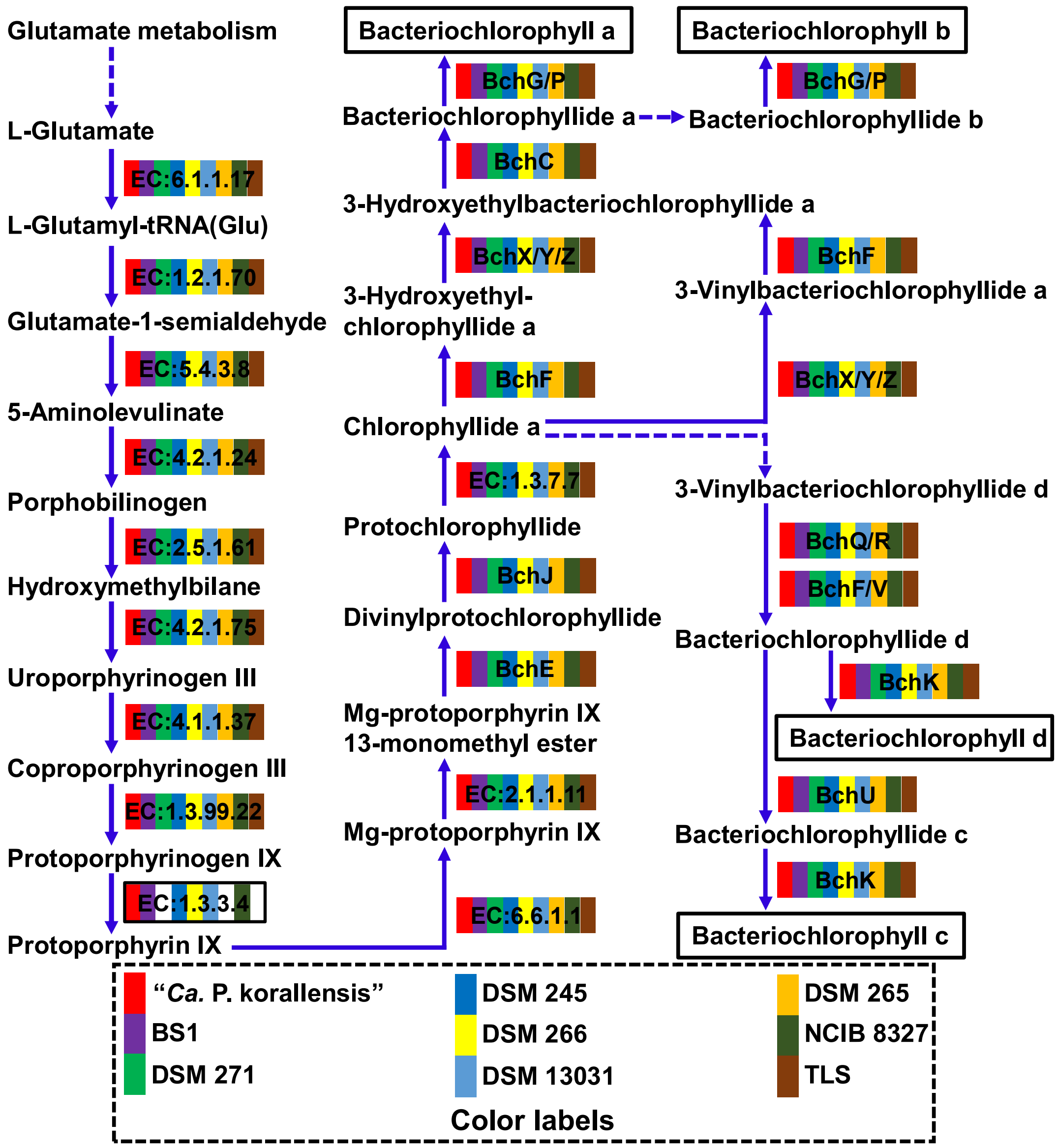

Figure S2 Bacteriochlorophyll biosynthesis pathway for "*Ca.* P. korallensis" and its relatives. Each color indicates a specific bacterium with certain function. Blank shows lack of certain function.

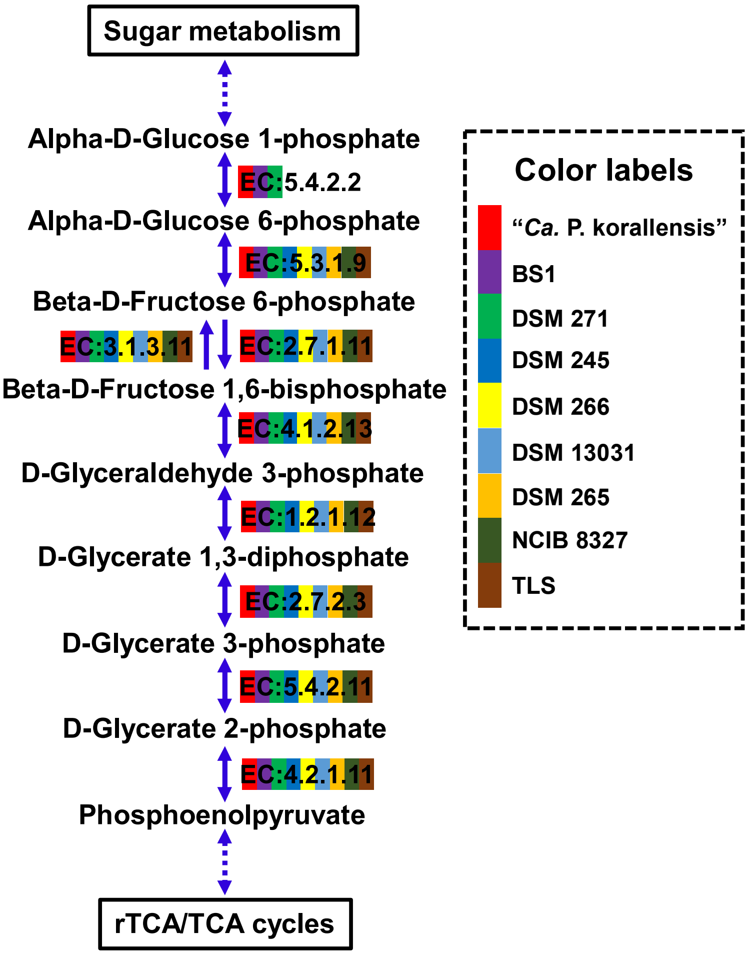

Figure S3 Gluconeogenesis and glycolysis pathway for "*Ca.* P. korallensis" and its relatives. Each color indicates a specific bacterium with certain function. Blank shows lack of certain function.

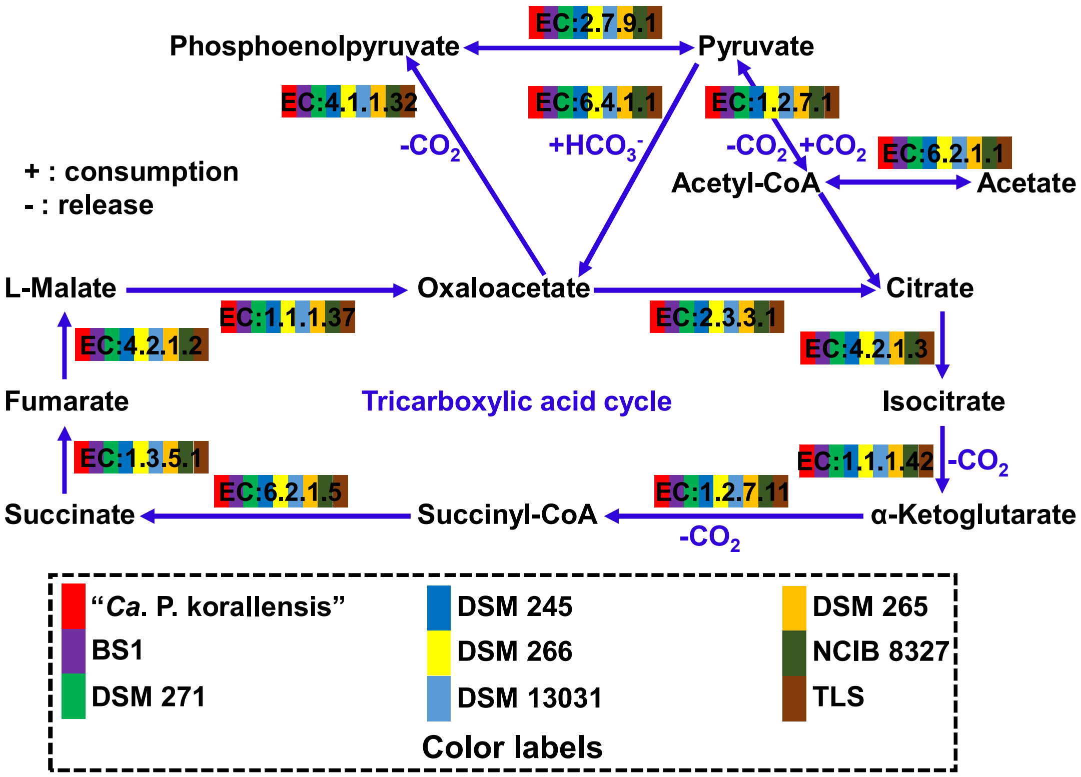

Figure S4 TCA cycle for "*Ca.* P. korallensis" and its relatives. Each color indicates a specific bacterium with certain function. Blank shows lack of certain function.

Table S1 Information of sampling locations, coordinates, dates, and species. Six colonies of each species were collected in each sampling.

Location	Lamma Island	Crescent Bay
Coordinate	E114.135°, N22.187°	E114.314°, N22.531°
Date	19-03-14, 21-10-2014	24-03-2014, 15-10-2014
	Porites lutea	*Porites lutea*
Species	*Montipora venosa*	*Montipora peltiformis*
	Platygyra carnosa	*Galaxea fascicularis*

Table S2 Information of metagenome sequencing datasets before and after quality control. "-1" and "-2" indicate the paired-end read 1 and 2, respectively.

Datasets	Raw reads	Clean reads	Clean reads %	Raw bases	Clean bases	Clean bases %
PC1-1	14964061	14160077	94.63%	1870507625	1721775362	92.05%
PC1-2	14964061	14160077	94.63%	1870507625	1712757579	91.57%
PC2-1	12486805	11867307	95.04%	1560850625	1441992037	92.39%
PC2-2	12486805	11867307	95.04%	1560850625	1438232485	92.14%
PC3-1	11305376	10571460	93.51%	1413172000	1280674352	90.62%
PC3-2	11305376	10571460	93.51%	1413172000	1273863970	90.14%
PC4-1	13077902	12338508	94.35%	1634737750	1500175586	91.77%
PC4-2	13077902	12338508	94.35%	1634737750	1492411643	91.29%
PC5-1	11686678	11078033	94.79%	1460834750	1342176138	91.88%
PC5-2	11686678	11078033	94.79%	1460834750	1342399705	91.89%
PC6-1	10963568	10378336	94.66%	1370446000	1257679792	91.77%
PC6-2	10963568	10378336	94.66%	1370446000	1256554525	91.69%
PC7-1	10298960	9767117	94.84%	1287370000	1183467286	91.93%
PC7-2	10298960	9767117	94.84%	1287370000	1183695147	91.95%
PC8-1	13369064	12698100	94.98%	1671133000	1540406249	92.18%
PC8-2	13369064	12698100	94.98%	1671133000	1537077315	91.98%
PC9-1	13447490	12729132	94.66%	1680936250	1539722942	91.60%
PC9-2	13447490	12729132	94.66%	1680936250	1540755492	91.66%
PC10-1	15378039	14441981	93.91%	1922254875	1747505895	90.91%
PC10-2	15378039	14441981	93.91%	1922254875	1742672678	90.66%
PC11-1	14163508	13204970	93.23%	1770438500	1599768876	90.36%
PC11-2	14163508	13204970	93.23%	1770438500	1591136533	89.87%
PC12-1	12109664	11392804	94.08%	1513708000	1380009379	91.17%
PC12-2	12109664	11392804	94.08%	1513708000	1376340132	90.93%
